# Simulated ischemia/reperfusion-induced p65-Beclin 1-dependent autophagic cell death in human umbilical vein endothelial cells

**DOI:** 10.1038/srep37448

**Published:** 2016-11-18

**Authors:** Min Zeng, Xin Wei, Zhiyong Wu, Wei Li, Yin Zheng, Bing Li, Xuqing Meng, Xiuhong Fu, Yi Fei

**Affiliations:** 1 Medical Center, Hainan General Hospital, Haikou, 570311, China

## Abstract

Myocardial ischemia/reperfusion (I/R) injury detrimentally alters the prognosis of patients undergoing revascularization after acute myocardial infarction. Our previous study demonstrated that NF-κB-induced autophagy plays a detrimental role in cardiac I/R injury using a rabbit myocardial I/R model. In this study, we sought to explore the specific mechanism of this autophagy-mediated cell damage in an *in vitro* simulated ischemia/reperfusion (sI/R) model using human umbilical vein endothelial cells. Our current study demonstrates that simulated I/R induces autophagy in a p65-Beclin 1-dependent manner, which can be suppressed with the inhibition of NF-κB. Furthermore, rapamycin which promotes autophagy, exacerbates sI/R-induced cell death. While 3-methyladenine rescues cell damage. Our data thus suggest that I/R promotes NF-κB p65 activity mediated Beclin 1-mediated autophagic flux, thereby exacerbating myocardial injury.

Autophagy is a mechanism of degradation of cytoplasmic components within an autophagosome, a characteristic double-membrane structure, which later fuses with lysosomes^1^,^2^. There are three types of autophagy: macro-autophagy, micro-autophagy, and chaperone-mediated autophagy^1^. Our use of the term “autophagy” in this article refers to macro-autophagy. Autophagy is controlled by autophagy-related genes (Atgs), many of which are involved in autophagosome formation^3^. For instance, Beclin1 (Atg6) and class III phosphoinositide 3-kinase (PI3K) are needed for the vesicle (called isolation membrane) nucleation step of autophagy. The vesicle elongation process features a conjugation system that is well-conserved among eukaryotes. The conjugation of phosphatidylethanolamine to Atg8 (microtubule-associated protein 1 light chain 3; LC3) by the sequential action of Atg4, Atg7 and Atg3 leads to the conversion of the soluble form of LC3 (LC3-I) to the autophagic vesicle-associated form (LC3-II), which is used as a marker of autophagy. Mounting evidence supports autophagy’s key role in cardiovascular disease such as cardiac hypertrophy, heart failure and myocardial ischemia/reperfusion (I/R) injury^4^,^5^. Although in some situations autophagy may function as a survival mechanism, excessive autophagy leads to cell death. Autophagy serves as a double-edged sword in cardiovascular disease, acting in either beneficial or maladaptive ways depending on the context^6^. Our previous study revealed that myocardial I/R activated NF-κB p65 to promote a no-reflow phenomenon in rabbits^7^. In addition, our *in vivo* experiments verified that autophagy mediated by p65 plays an important role in the mechanism of myocardial I/R injury.

NF-κB signaling has also been implicated in cardiac I/R injury^8^,^9^. P50-p65/RelA heterodimer, which is the major complex in most cells, is commonly referred specifically and hereinafter as NF-kB. Intracellular NF-κB resides inactive and bound to the inhibitory protein IκB in the cytoplasm. This complex is then activated by numerous stimuli. Increased activity of an IκB kinase (IKK) complex mediates the activation of NF-κB. The liberated heterodimer p50-p65/RelA translocates into the nucleus, where it activates downstream genes to fulfill its pathophysiologic function^10^. Beclin1 has also been implicated as one of these downstream genes. In a previous study, we verified a simulated ischemia/reperfusion (sI/R) system by combining hypercarbia with low pH, hyperkalemia, and glucose deprivation to mimic key aspects of ischemia and then replaced this with normal medium to simulate reperfusion^11^. Since endothelial cells are sensitive to subtle changes in blood supply such as hypoxia and energy deprivation and are convenient for manipulation and culture, we believe human umbilical vein endothelial cell (HUVEC) lines are a favorable model for the research of cardiac I/R pathophysiology. The aim of this study is to illuminate the associated pathway by which sI/R induced autophagy exerts effects on HUVECs. Knowledge of the role of key mediators in this pathway will help to indentify therapeutic targets for patient suffering from myocardial ischemia/reperfusion injury.

## Results

### sI/R promotes autophagic protein expression and autophagic flux in HUVECs

**Figure 1:**
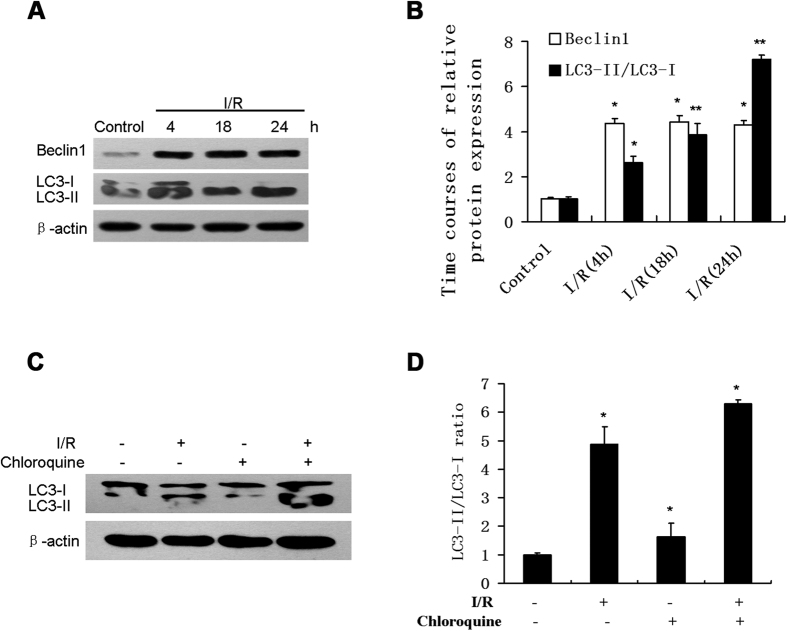
sI/R promoted Beclin 1 Protein and autophagic flux in HUVECs. (**A**) HUVECs exposed to normal culture media (control) or 30 min of “ischemic buffer” followed by switching to normal media for 4 h, 18 h and 24 h (I/R). Cell lysates were then subjected to immunoblot for Beclin1, LC3 B and β-actin. (**B**) Graph showing the quantification of the immunoreactive bands obtained as **(A)**. (**C**) HUVECs pretreated with or without chloroquine (3 μM) for 1 h were subjected to normal media or I/R for 4 h. Cell lysates were analyzed by Western blotting for detection of LC3 B and β-actin. (**D**) Graph showing quantification of LC3-I to LC3-II conversion from the immunoreactive bands obtained as (**C**). *P < 0.05 vs control. **P < 0.01 vs control.

sI/R resulted in a significant increase in Beclin1 protein levels, which occurred within 4 h of reperfusion and persisted for at least 24 h (Fig. 1A). Protein expression of Beclin1 in sI/R at 4 h, 18 h and 24 h were 4.36, 4.41, and 4.28 fold, respectively, compared to the control (Fig. 1B). When autophagy is activated, cytosolic LC3-I is cleaved to proteolytic derived LC3-II. Thus, we used detection of LC3-II to estimate the abundance of autophagosomes. After “ischemia” for 30 min, a reperfusion time-dependent activation of autophagy was observed in HUVECs as measured by the conversion of LC3-I to LC3-II (Fig. 1A). sI/R-induced increases in LC3-II/LC3-I ratio at 4 h, 18 h and 24 h were 2.61, 3.87, and 7.20 fold, respectively, compared to the control (Fig. 1B). However, we had concerns that an increase in LC3-II/LC3-I ratio might not represent induction of autophagy and might instead be due to I/R repressing autophagosome fusion with lysosomes and degradation of LC3-II. Therefore, in order to determine the flux of autophagy, which reflects true autophagic activity, we carried out an LC3 turnover assay by examining the effect of disrupting lysosomal function using lysosomotropic reagent chloroquine (Fig. 1C). Chloroquine increases lysosomal pH and blocks the degradation of LC3-II, resulting in the accumulation of LC3-II. When treated with chloroquine, I/R-stimulated conversion of LC3-I to LC3-II in HUVECs increased by 28.95% more than I/R alone (Fig. 1D) (p < 0.05).

### sI/R-induced autophagy is NF-κB p65-dependent

**Figure 2:**
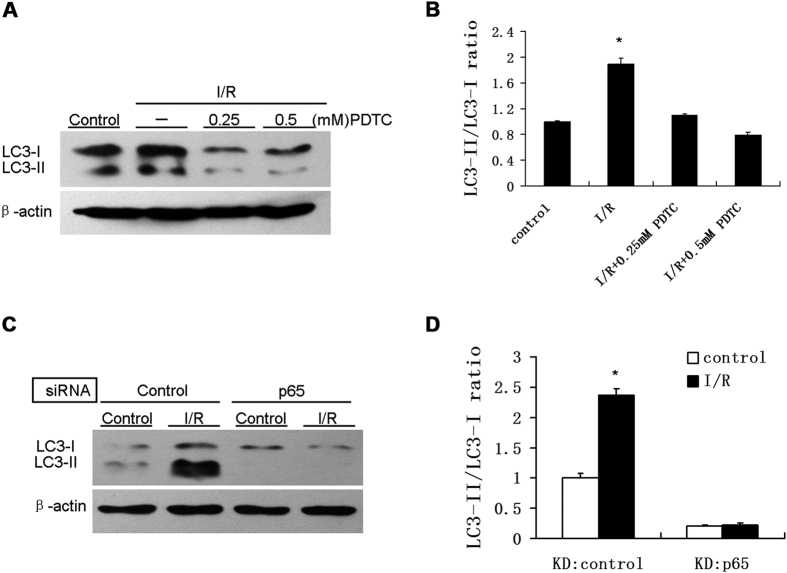
sI/R-induced LC3-I to LC3-II conversion was p65 dependent. (**A**) HUVECs were pretreated with 0.25 mM or 0.5 mM PDTC for 1 h before exposed to normal media (control) or I/R for 4 h. Cell lysates were subjected to immunoblotting analysis for detection of LC3B. (**B**) Graph showing quantification of LC3-I to LC3-II conversion from the immunoreactive bands obtained as (**A**). *P < 0.05 vs control. (**C**) HUVECs were transfected with control siRNA or p65 siRNA oligos (each at 20 nM) before exposure to normal media (control) or I/R conditions for 4 h. Immunoblotting with LC3 antibody was then performed. (**D**) Quantification of LC3-I to LC3-II conversion was measured from the immunoreactive bands obtained as (**C**), normalized to those of β-actin and the data were presented as a mean ± SD from three independent experiments. *P < 0.05 vs control.

To determine whether p65 was involved in the sI/R activation of autophagy, we pretreated HUVECs with pyrrolidine dithiocarbamate (PDTC)^7^,^12^, the specific NF-κB inhibitor, for 1 h prior to sI/R exposure. The sI/R-induced conversion of LC3-I to LC3-II was reduced by PDTC in a dose-dependent manner. 0.25 mM and 0.5 mM of PDTC decreased sI/R-induced LC3-II/LC3-I ratio by 42.10% and 58.42% (Fig. 2A,B). To circumvent potential side effects of the inhibitor, p65 siRNA was transfected into HUVECs to substantiate the role of NF-κB. Consistently, knocking down of p65 dramatically reduced I/R-induced LC3II/LC3I ratio by 90.72% compared to cells transfected with a scrambled control siRNA (Fig. 2C,D) (p < 0.05).

### sI/R-induced development of autophagic vacuoles is NF-κB p65-dependent

**Figure 3:**
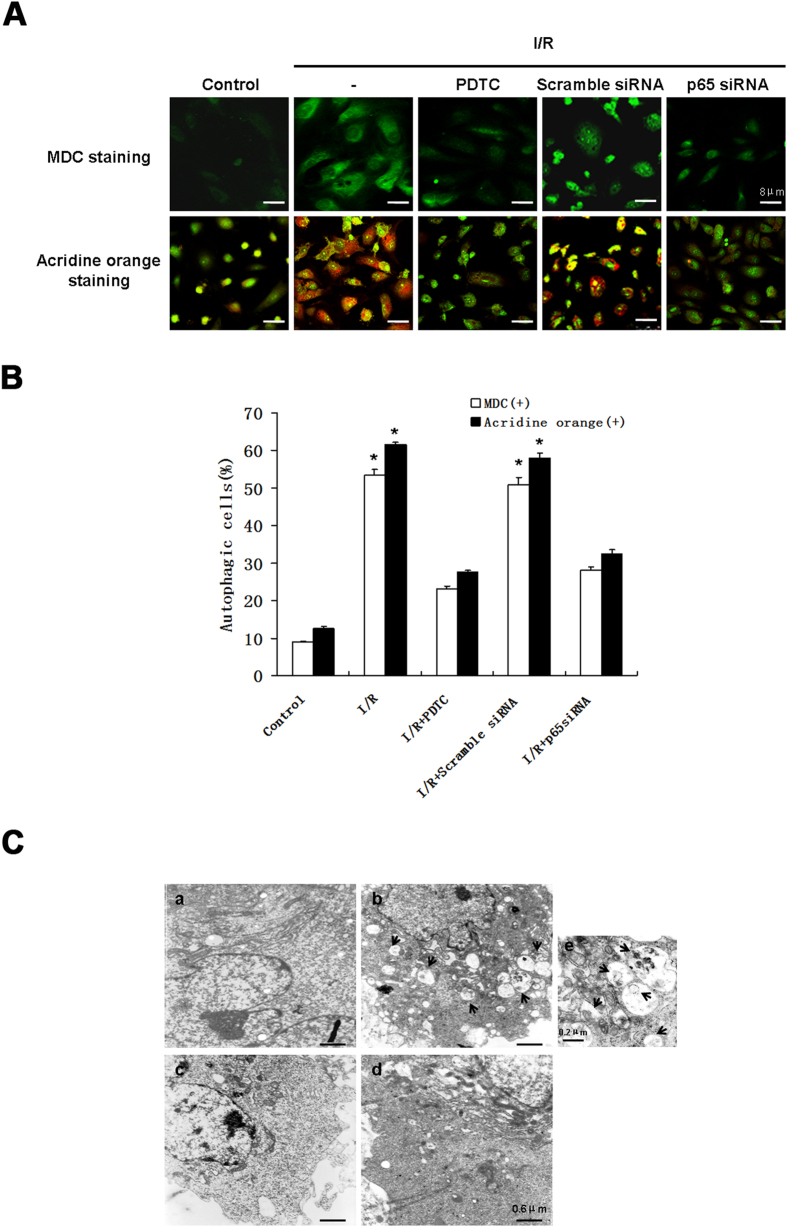
Suppression of NF-κB p65 by PDTC or p65 siRNA diminished sI/R-induced development of autophagic vacuoles. (**A**) HUVECs subjected to normal media (control) or I/R for 4 h after either transfected with a scramble or p65 siRNA for 48 h or treated with 0.5 mM PDTC for 1 h, were labeled either with 50 μM MDC (upper) or 10 μM acridine orange (lower). Note the formation of green (upper) or red particles (lower)–accumulating autophagic vacuoles in I/R treatment cells (Confocal microscopy, Bar, 8 μm.). (**B**) The percentage of cells with MDC-stained dots and acridine orange–accumulating autophagic cells was quantified. Representative results of three independent experiments. *P < 0.05. **(C**) HUVECs in presence (c,d) or absence (a,b) of p65 siRNA for 48 h before subjected to normal media (a and c, control) or I/R (b and d) for 4 h. a–d, representative electron micrographs of each sample were shown. Bar, 0.6 μm. e, higher magnification of I/R-induced autophagosomes containing damaged organelles. Bar, 0.2 μm. Arrowheads indicated autophagic vacuoles.

We examined the effect of I/R injury on the formation of autophagic vacuoles in HUVECs as measured by Monodansylcadaverine (MDC) or acridine orange staining. MDC is a fluorescent dye used to detect autophagic vacuoles, the accumulation of mature autophagic vacuoles such as autophagosomes rather than the early endosomal compartment^13^. With MDC staining, bright green dots representing autophagosomes in the cytoplasm of HUVECs increased in our stimulated sI/R model compared to the control. I/R-induced autophagosomes were decreased 47.17% and 56.60% by knockdown of p65 and PDTC(0.5 mM) treatment, respectively (Fig. 3A, upper panel and Fig. 3B). As an alternative method, using acridine orange stain, the protonated form of acridine orange accumulated in acidic compartments was characterized by red fluorescence. In the control group, cells displayed primarily green fluorescence with minimal red fluorescence indicating the absence of autophagic vacuoles, while exposure of HUVECs to sI/R produced a 4.87-fold increase in red fluorescence. This red fluorescence decreased by 47.58% with transfection of p65 siRNA and by 55.13% through PDTC treatment (Fig. 3A, lower panel and Fig. 3B). However, we were concerned about that neither MDC nor acridine orange staining is specific for autophagosomes; vesicles with positive staining may be indiscernible from lysosomes. Therefore, we additionally used electron microscopy (EM) to visualize autophagic vacuoles, which has been considered a reliable method for identifying autophagosomes (Fig. 3C,a–e). EM revealed a consistent increase in the number of autophagic vacuoles containing degraded cytoplasmic materials after sI/R (Fig. 3C,b). Silencing of p65 by siRNA diminished the phenomenon observed above in sI/R (Fig. 3C,d).

### sI/R-induced expression of Beclin1 is mediated by NF-κB p65

**Figure 4:**
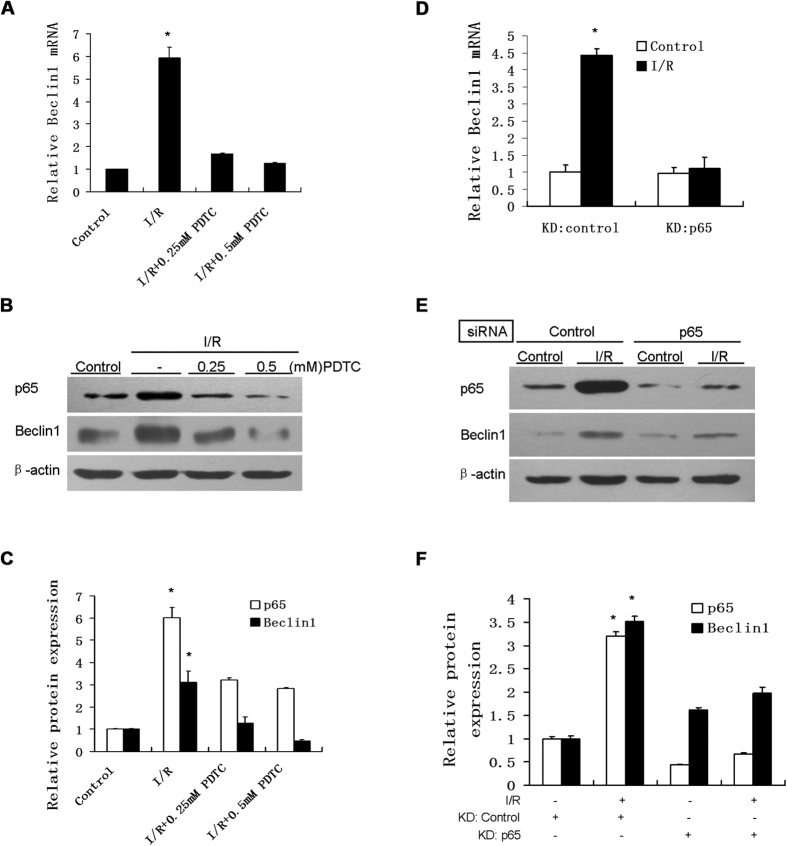
PDTC inhibited sI/R-induced expression of Beclin1. HUVECs were pretreated with 0.25 mM or 0.5 mM PDTC for 1 h before exposed to normal media (control) or I/R for 4 h. (**A**) Total RNA was extracted to measure Beclin1 mRNA level by real time quantitative reverse transcription-PCR. Values obtained for Beclin1 mRNA transcripts were normalized to those of β-actin. *P < 0.05. (**B**) Cell lysates were subjected to immunoblot analyses for detection of p65, Beclin1 and β-actin. (**C**) The immunoblot bands from (**B**) corresponding to the Beclin1 and p65 protein levels were quantified using ImageJ software and normalized to β-actin. (**D**) HUVECs were transfected with scramble (control)siRNA or p65 siRNA oligos (each at 20 nM) before exposure to normal media (control) or I/R conditions for 4 h. Beclin1 mRNA level was quantified by real-time PCR. Values obtained for Beclin1 mRNA transcripts were normalized to those of β-actin. *P < 0.05 versus control and KD p65 in I/R. (**E**) Immunoblotting with the indicated antibodies was performed. (**F**) Densitometry values of bands from (**E**) for Beclin1 and p65 protein levels were expressed as fold change compared with β-actin and normalized to 1. Results shown in A-D are representative of three independent experiments.

Since activation of p65 correlated with increased Beclin1 expression in the context of I/R injury both *in vivo*^7^ and *in vitro*^11^, we sought to determine whether p65 is upstream of Beclin1 in the cell signal transduction pathway of sI/R-induced autophagy. Pretreatment of HUVECs with 0.25 mM and 0.5 mM of PDTC in sI/R resulted in 71.84% and 78.41% reduction of Beclin1 mRNA respectively compared to sI/R treatment alone as detected by real time PCR (Fig. 4A). This effect was dose-dependent, with a maximal effect seen at 0.5 mM. We next investigated the effect of PDTC on protein levels of Beclin1 by Western blot. Treatment with 0.25 mM and 0.5 mM of PDTC decreased I/R-induced protein levels of Beclin1 by 59.62% and 84.86% respectively (Fig. 4B,C). To confirm the effect of p65 on Beclin1 in sI/R, we specifically knocked down endogenous p65 in HUVECs via RNA interference. Knockdown of p65 led to a 75.11% reduction in I/R-induced Beclin1 mRNA levels (Fig. 4D). Furthermore, in HUVECs with p65 siRNA transfection in sI/R, we measured a 79.06% reduction of p65 protein levels followed by a 43.43% reduction of I/R-induced Beclin1 protein levels (Fig. 4E,F).

### Rapamycin threatens cell survival while 3-MA promotes cell survival in the context of sI/R

**Figure 5:**
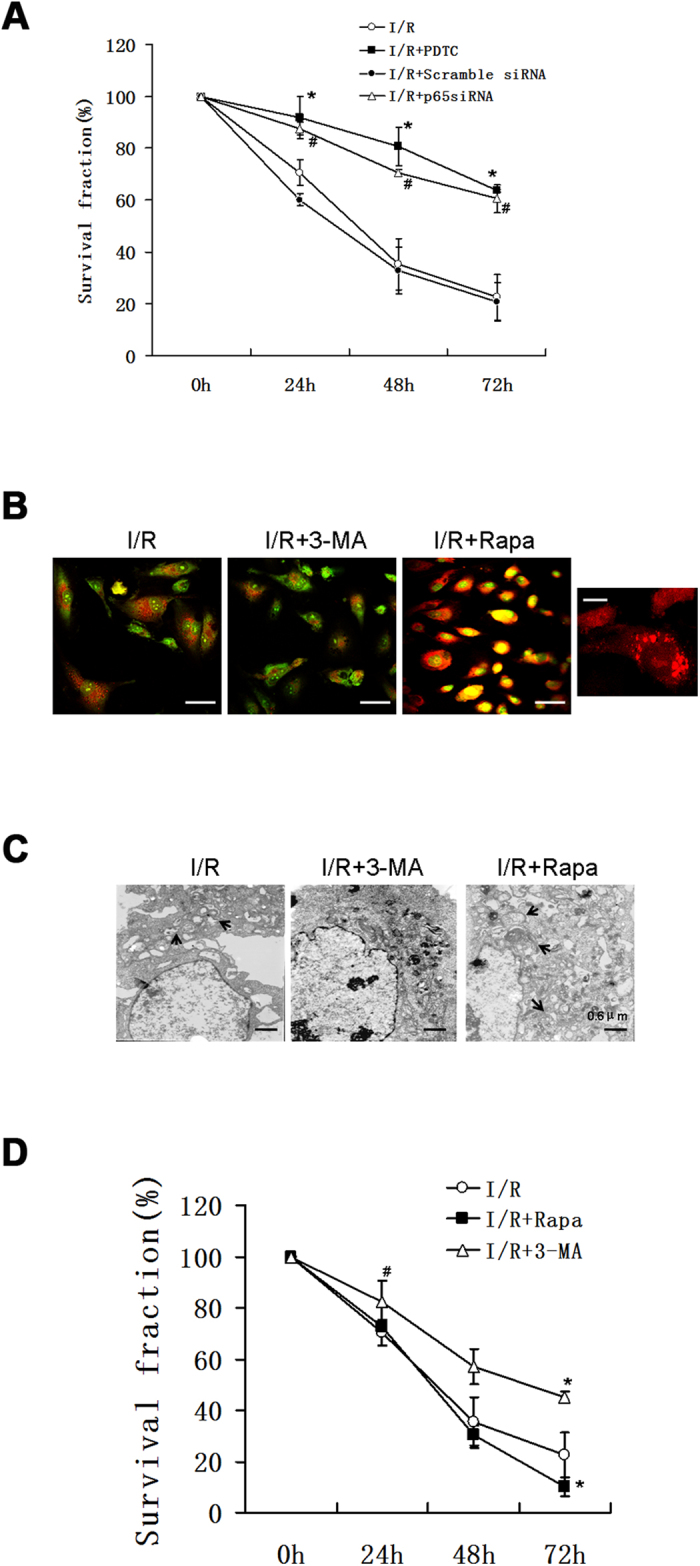
Rapamycin deteriorated cell death, but 3-MA promoted cell survival in sI/R. (**A**) HUVECs were cultured in ischemic buffer for 30 minutes followed by normal media in the absence or presence of PDTC (0.5 mM) or transfection of p65 siRNA(20 nM) or scramble siRNA(20 nM). WST-1 assay was conducted to evaluate cell vitality. HUVECs subjected to ischemic buffer for 30 minutes followed by normal media for 4 h in the absence or presence of rapamycin (0.5 mM) or 3-MA (10 mM), were examined under confocal microscopy with 10 μM acridine orange (**B**) (Bar, 8 μm. Left, maglification, Bar, 5 μm.) or under EM (**C**) (Bar, 0.6 μm. Arrowheads indicated autophagic vacuoles.) or for WST-1 assay to evaluate cell vitality (**D**). ^#^P < 0.05; *P < 0.01. All the experiments were conducted in triplicate at least three times.

WST-1 cell viability assays revealed that sI/R induced cell death in a time-dependant manner by 29.55%, 64.78% and 77.54% at 24 h, 48 h and 72 h (Fig. 5A). To verify whether p65 plays a role in sI/R-related reduction in cell survival, we assessed the effects of PDTC and p65 siRNA under sI/R conditions. PDTC (0.5 mM) treatment significantly decreased sI/R-induced cell death by 72.28%, 70.27% and 53.07% at 24 h, 48 h and 72 h. Correspondingly, p65 siRNA (20 nM) treatment significantly reduced sI/R-induced cell death by 57.97%, 54.31% and 49.05% at 24 h, 48 h and 72 h. In addition, because 3-MA is a well-known inhibitor of autophagy and rapamycin is a well-known stimulator of autophagy, we confirmed the effects of 3-MA and rapamycin on the formation of autophagic vacuoles in our sI/R system with acridine orange staining under confocal laser microscopy. Exposure of HUVECs to 3-MA in sI/R exhibited rather faint perinuclear red fluorescence compared to sI/R alone (Fig. 5B). On the contrary, HUVECs pretreated with rapamycin glowed with bright red perinuclear fluorescence. These red dots were distinct and countable under magnification. Furthermore, HUVECs pretreated with 3-MA ahead of sI/R displayed an absence of autophagic vacuoles under EM, while we observed quite the opposite phenomenon in cells pretreated with rapamycin (Fig. 5C). Cell viability assays revealed that 3-MA treatment significantly decreased sI/R-induced cell death by 29.30% at 72 h. Rapamycin treatment significantly promoted sI/R-induced cell death by 15.88% at 72 h with no statistical difference detected at 24 h and 48 h (Fig. 5D).

## Discussion

**Figure 6:**
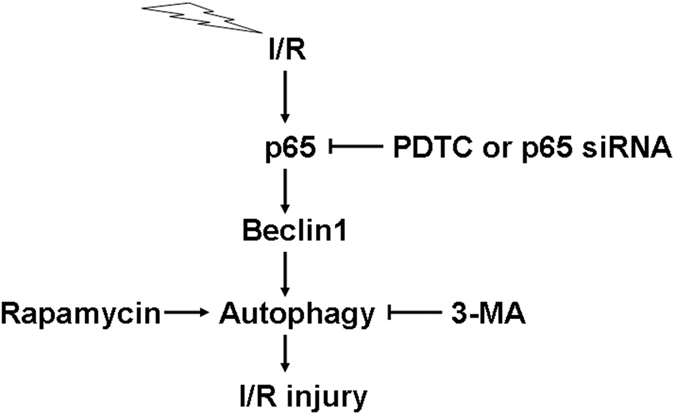
Schematic representation of a novel pathway for the induction of autophagy in response to sI/R. sI/R induced NF-κB p65-mediated expression of Beclin1 and autophagy. Intemperate autophagy aggravated sI/R-induced cell injury.

Complications such as malignant arrhythmia, heart failure and other cardiovascular events resulting from ischemia-reperfusion injury are not rare in patients undergoing revascularization after acute myocardial infarction. Elucidating the mechanisms behind myocardial ischemia-reperfusion injury is critical in achieving a better clinical prognosis for these patients. Besides the roles of apoptosis, endoplasmic reticulum stress, reactive oxygen species and so on, autophagy has been implicated as contributing to the ischemia-reperfusion injury process. It is also known that NF-κB plays a crucial role in ischemia-reperfusion injury. However, the downstream pathway is not clearly understood. In the present study, we provide evidence of a novel pathway for the induction of autophagy in response to sI/R. sI/R induced NF-κB p65-mediated expression of Beclin1 and autophagy (Fig. 6).

During the formation of autophagosomes, Beclin 1, a mammalian ortholog of yeast Atg6, plays a very important role in the vesicle nucleation step of autophagy, and therefore serve as a marker for autophagy. Elevated levels of Beclin 1 protein were associated with an increase in autophagy phenomenon^14^,^15^. It is reported that Beclin 1-dependent autophagy mediates autophagic cell death during I/R^16^,^17^. Reduction of I/R-induced autophagy by urocortin or RNA interference-reduced Beclin 1 expression in cardiac myocytes enhanced cell survival^18^. An induction of autophagy and cardiac injury during the reperfusion phase was significantly attenuated in Beclin +/− mice^19^. However, an increase in Beclin 1 protein levels alone does not absolutely guarantee an increase in autophagy. Binding of Beclin 1 with Bcl-2 family proteins was reported to negatively regulate autophagy activity, in response to some external stimuli^15^. For example, Beclin 1-mediated autophagy implicated to play a protective role in preconditioning. Autophagy elicited by ischemic preconditioning inhibited necrosis in injured livers. Enhancement of Bcl-2 phosphorylation was associated with decreased interactions between Bcl-2 and Beclin 1 and an increased expression of microtubule-associated protein 1 light chain 3 (LC3)-II, suggesting important roles of Bcl-2 and Beclin 1 in the regulation of autophagy^20^,^21^. Since LC3-II is essential for autophagosome formation, the conversion ratio of LC3-II/LC3-I is widely used to monitor autophagic activity. Our present study demonstrates that Beclin 1 protein levels were increased after 4 h in sI/R and remained steadily at high levels at 24 h afterwards in sI/R. LC3-I to LC3-II conversion continued rising in a time-dependent manner in sI/R. Based on these results, we propose that Beclin 1 expression responds quickly to sI/R and is the main, but probably not the only, mediator to trigger activation of autophagy in sI/R. Moreover, in some cases, increased expression of Beclin-1 and LC3-II may not represent enhanced autophagic activity, but rather as a result of reduced degradation of autophagic vacuoles which results in either feedback stimulation of Beclin-1 or accumulation of existing LC3-II^22^. Ma, X *et al*. reported that impaired autophagosome clearance because of lysosomal activity inhibition contributed to cardiomyocyte death in I/R injury^23^. Therefore, it is important to distinguish autophagic flux from steady-state level of LC3 monitoring. The latter may be assessed by following LC3 in the absence and presence of autophagy inhibitors, and by examining the autophagy-dependent degradation of appropriate substrates^24^. In particular, with the evidence of an increase in LC3-II and autophagosomes in the present study, it is essential to determine whether this represents increased flux, or a block in fusion or degradation using inhibitors such as chloroquine. Chloroquine disrupts lysosomal function by increasing lysosomal pH and blocks the degradation of LC3-II, resulting in the accumulation of LC3-II. Treatment with chloroquine hence helps to determine autophagic activity, which represents the amount of LC3 delivered to lysosomes for degradation (i.e., autophagic flux). In our present study, inhibition of lysosomal activity with chloroquine further enhanced the level of LC3-II in HUVECs subjected to sI/R. Therefore, we have confirmed that sI/R not only promotes autophagic protein expression but also autophagic flux in HUVECs. Correspondingly, I/R promotes autophagic activity and causes autophagic cell death in H9c2 cells^25^. In support of our findings, based on their *in vivo* research, Yu, P *et al*. reported the protection effect of sevoflurane postconditioning in myocardial I/R through inhibition of over-activation of autophagy by suppressed elevated expressions of LC3 II/I ratio, Beclin1, Atg5 and Atg7 in I/R rat^26^.

Transcription factor NF-κB is an important mediator to stimulate downstream genes in response to environment stimulation outside cells. NF-κB has been implicated in restricting autophagy in a positive^27^ or negative way^28^,^29^ in different scenarios. We hypothesize that sI/R-induced autophagy might involve the stimulation of gene spectrum such as NF-κB. Pretreatment with PDTC and p65 siRNA transfection to suppress NF-κB both lead to the inhibition of LC3-II expression or the disruption of autophagosome development. Our previous study indicated that NF-κB-induced autophagy played a pivotal role in rabbit myocardial I/R injury. Our current *in vitro* study confirms that sI/R promotion of autophagosome formation as well as autophagic activity is mediated by NF-κB. In support of our findings, inhibition of NF-κB associated autophagy showed a protective effect against I/R injury in the brains of rats^30^. In addition, Shen M *et al*. reported ethyl pyruvate attenuates hepatic I/R injury by inhibiting the intrinsic pathway of autophagy mediated partly through downregulation of the NF-κB axis^31^.

p65/RelA has consensus sites in the promoter with the Beclin 1 gene and p65/RelA positively modulates canonical autophagy in various human cell lines^32^. Evidence that beclin-1 is NF-κB regulated has been previously published^33^. Furthermore, it is reported that the expression of Beclin1 is dependent upon NF-κB^34^ and inhibition of NF-κB suppresses autophagy. However, a decrease in phosphorylation of IκBα and NF-κB/p65 and NF-κB/p65 nuclear translocation was correlated with upregulation of Beclin 1 in SZC014-induced autophagy^35^. The research of Rui, L.X. *et al*. suggested that SZC014, a synthetic oleanolic acid derivative, induced autophagy in gastric cancer cells via NF-κB pathway. Many mechanisms have been implicated that involve Beclin 1 participating in autophagy during I/R injury. It is tempting to speculate whether NF-κB is involved in the Beclin 1 associated I/R-induced autophagy. Manipulation of wild type or transgenic IκBα dominant-negative mice unveiled that Beclin-1 and LC-3 are elevated in a NF-κB-dependent manner in the setting of myocardial I/R^36^. For the first time, our study suggested that Beclin 1 is downstream of NF-κB in the context of sI/R. The observed increase in autophagy is NF-κB-dependent, and likely includes direct regulation of beclin-1 mRNA and protein levels.

Whether autophagy is beneficial or detrimental to cells remains controversial^37^. For example, down-regulation of miRNA-30a alleviated cerebral ischemic injury through enhancing Beclin 1-mediated autophagy^38^. Facilitating the formation of autophagolysosomes protects the heart against second hand smoke exposure-induced myopathic changes^39^. Evidence supports that autophagy benefits heart cells during myocardial ischemia through improving myocardial energy metabolism and organelle recycling^40^,^41^. In agreement with these findings, delayed anesthetic preconditioning protected rat hearts from I/R injury via activation of NF-κB and upregulation of autophagy^42^. In addition, NF-κB-dependent increase in autophagy is indispensable in cardioprotection as ischemic preconditioning against I/R injury by acute high-fat feeding^36^. However, autophagy actually is one of the major types of morphologically distinct cell death, called typy II cell death^43^. Excessive autophagy produces lethal damage on cells^44^, confirmed by data showing that inhibition of excessive autophagy in cardiomyocytes reduces cardiac ischemia/reperfusion injury^5^. Specifically, in the context of sI/R, 3-MA rescues HUVECs by inhibiting autophagy while rapamycin promotes cell death by enhancing autophagy. Indeed, autophagy has great influence over a cell’s decision to live or die. Our data support the hypothesis that sI/R threatens cell survival by stimulating NF-κB dependant autophagy.

In brief, the present study reveals that Beclin 1-associated autophagy promotes cell death in sI/R. Activation of NF-κB is a plausible mechanism responsible for this effect. These findings suggest the possibility that inhibition of excessive autophagic response could prevent I/R-induced myocardial injury.

### Study limitations

It is worth to point out that the limitation exists in this study. All our experiments were conducted in HUVECs. For the deference between venous endothelial cells and arterial endothelial cells, our results from HUVECs might not completely translate to arterial endothelial cells.

## Methods

### Cell culture

Human umbilical vein endothelial cells (HUVECs) were purchased from ScienCell Research Laboratories (Carlsbad, CA, USA) and cultured in Endothelial Cell Medium (ECM) supplemented with endothelial cell growth supplement (ECGS), 5% fetal bovine serum (FBS) and penicillin/streptomycin (P/S) solution (ScienCell, Carlsbad, CA, USA) in an incubator at 37 °C with 5% CO_2_. Cells of passage No. 4–6 were grown until confluence for experiments. Either pyrrolidine dithiocarbamate (PDTC) at concentrations of 0.25 mM and 0.5 mM or chloroquine (3 mM) were added 1 h prior to the exposure of HUVECs to normal conditions or simulated I/R.

### HUVECs simulated ischemia/reperfusion (sI/R) protocol

sI/R was initiated by incubation of HUVECs for 30 min in an “ischemic buffer” containing 118 mM NaCl, 24 mM NaHCO_3_, 1.0 mM NaH_2_PO_4_, 2.5 mM CaCl_2_, 1.2 mM MgCl_2_, 20 mM sodium lactate, 16 mM KCl, and 10 mM 2-deoxyglucose (pH adjusted to 6.2), followed by “reperfusion” for 4 h. “Reperfusion” was accomplished by replacing the ischemic buffer with normal medium and culturing cells under normoxic conditions. We incorporated 2-deoxyglucose in our protocol to prevent cells from using their large glycogen reserves and thus truly simulate the substrate deprivation of ischemia.

### Reagents

The antibodies used in this study were obtained from the following manufacturers: Beclin1 and LC3B from Cell Signalling Technology (Beverly, MA); NF-κB p65 and β-Actin from Santa Cruz Biotechnology (Santa Cruz, CA); rapamycin from LC laboratories. Other Chemicals including Acridine orange, Monodansylcadaverine (MDC), chloroquine, 3-methyladenine (3-MA) and PDTC were all from Sigma.

### Real Time PCR Assay

RNA was extracted using trizol reagent (Invitrogen), and quantitative reverse transcription PCR was performed using the iScript TM cDNA Synthesis kit (BIO-RAD). 18 S rRNA served as an endogenous control. We used a SYBR Premix Ex Taq (Applied TaKaRa) and iQ5 Real-time PCR detection system (BioRad) for real-time quantification. Primer sequences were 5′-AGGTTGAGAAAGGCGAGACA-3′ and 5′- AATTGTGAGGACACCCAAGC-3′ for Beclin-1, and 5′-CGGCTACCACATCCAAGGAA-3′ and 5′-GCTGGAATTACCGCGGCT-3′ for 18 S rRNA.

### *In vitro* siRNA transfection

Pre-designed and validated siRNA specific for NF-κB p65, and non-targeting siRNA for control were purchased from Santa Cruz (sc-29410). HUVECs were plated to reach 30–50% confluence on the day of transfection. 20 nM siRNA duplexes were introduced with Lipofectamine RNAiMAX (Invitrogen).

### Western blotting

HUVECs after treatment were lysed in chilled lysis buffer with protease inhibitor cocktail (Roche). BCA assay (Pierce) with bovine serum albumin as a standard was utilized to determine protein concentrations, and clarified lysates were boiled in SDS sample buffer. Samples were separated by 10% or 15% SDS-PAGE and transferred onto a polyvinylidene difluoride (PDVF) membrane (Millipore). Membranes were blocked in 5% non-fat milk in Tris-buffered saline (TBS) containing 0.1% Tween 20 (TBS-T) for 1 h at room temperature, incubated overnight with relevant antibodies at 4 °C, washed, probed with species-specific secondary antibodies coupled to horseradish peroxidase, and visualized using ECL chemiluminescence.

### Immunofluorescent confocal laser microscopy

Autophagy is characterized by the development of autophagic vacuoles. Monodansylcadaverine (MDC) has been proposed as a tracer for autophagic vacuoles as described previously^11^. HUVECs were cultured on poly-L-lysine-coated cover slips overnight, then treated with different stimuli as described above, and rinsed with PBS. They were then stained with 50 μM MDC at 37 °C for 1 h. After incubation, the cells were fixed for 15 min with ice-cold 4% paraformaldehyde at 4 °C, washed twice with PBS. Images were viewed and captured blindly by two observers using Leica TCS SP5 confocal laser microscopy. The formation of acidic vesicular organelles is related to cell autophagy^45^. Acridine orange–stained cells fluoresce diffuse green fluorescence, whereas the acidic compartments including autophagic vacuoles show bright red. Cells treated by different stimuli were incubated for 15 min with acridine orange (10 μM in medium from a 10 mM stock in water), washed with PBS, and then examined under Leica TCS SP5 confocal laser microscopy.

### WST-1 Cell viability assays

WST-1 cell reagent is a simple, colorimetric assay for use in measuring cell viability^46^. The assay principle is based on the conversion of the tetrazolium salt WST-1 into a colored dye by mitochondrial dehydrogenase enzymes. The soluble salt is released into the media. Within a given time period, the reaction produces a color change which is directly proportional to the amount of mitochondrial dehydrogenase in a given culture. As a result, the assay actually measures the net metabolic activity of cells. WST-1 Cell Proliferation and Cytotoxicity Assay Kit (Beyotime institute of Biotechnology) was used to assess the cell viability of the HUVECs. HUVECs were plated at 7500 cells/well into 96-well tissue culture plates and then were allowed to attach for 12 hours. Then the media was switched to ischemic buffer for 30 minutes followed by replacement with normal media in the absence or presence of rapamycin (0.5 mM) or 3-MA (10 mM) or PDTC (0.5 mM) or p65 siRNA (20 nM). WST-1 reagent was added in 24 hour increments up to 72 hours, and absorbance was recorded at 450 nm wavelength following the manufacturer’s instructions. The experiments were conducted in triplicate three times.

### Electron microscopy

Electron microscopic analysis was performed as published previously^7^, with minor modifications. Briefly, HUVECs after treatment were fixed for 30 min with ice-cold 3% glutaraldehyde in 0.1 M cacodylate buffer, embedded in Epon, and processed for transmission electron microscopy using standard procedures. Representative areas were chosen for ultra-thin sectioning and examined on transmission electron microscope at X6, 000 magnification.

### Statistical analysis

Data were expressed as mean ± SD. Differences between two groups were analyzed using Student’s *t*-test. The data from more than two groups were evaluated by one-way ANOVA followed by the Newman-Keuls test. P < 0.05 is considered statistically significant.

## Additional Information

**How to cite this article**: Zeng, M. *et al*. Simulated ischemia/reperfusion-induced p65-Beclin 1-dependent autophagic cell death in human umbilical vein endothelial cells. *Sci. Rep*. **6**, 37448; doi: 10.1038/srep37448 (2016).

**Publisher’s note:** Springer Nature remains neutral with regard to jurisdictional claims in published maps and institutional affiliations.
